# Evaluation of water and electrolytes disorders in severe acute diarrhea patients treated by WHO protocol in eight large hospitals in Tehran; a nephrology viewpoint

**DOI:** 10.15171/jrip.2017.21

**Published:** 2016-11-24

**Authors:** Alireza Soleimani, Fatemeh Foroozanfard, Mohammad Reza Tamadon

**Affiliations:** ^1^Department of Internal Medicine, Kashan University of Medical Sciences, Kashan, Iran; ^2^Department of Gynecology and Obstetrics, Kashan University of Medical Sciences, Kashan, Iran; ^3^Department of Internal Medicine, Semnan University of Medical Sciences, Semnan, Iran

**Keywords:** Acute diarrhea, Water and electrolyte disorders, Acute renal failure, Acute kidney injury

## Abstract

**Introduction:** The most common cause of death from diarrhea is the shock caused by dehydration, electrolytes and acid-base disorders.

**Objectives:** The aim of this study was to evaluate water and electrolytes disorders in diarrhea patients after treating severe acute diarrhea.

**Patients and Methods:** In this study we used a historical cohort and studied patients who were hospitalized due to acute diarrhea and were similarly treated for dehydration and water and electrolyte disorders as recommended by the World Health Organization (WHO) guideline. Electrolytes, pH, serum creatinine (Cr) level on admission and during treatment were recorded. Patients with underlying diseases were excluded from the study.

**Results:** Of 121 patients who were enrolled in the study, 67.8% had hyponatremia on admission (plasma Na <137 mEq/L) and 5.8% had hypernatremia. Around, 33.88% of patients had hypokalemia and 2.4% had hyperkalemia. All hyperkalemia disorders were treated, but 87.1% of patients had hypokalemia or low potassium levels, or they were affected by uncorrected hypokalemia and were in need of further measures. Of all, 56.75% had acidosis and 21% of patients with acidosis were not treated or the severity of their acidosis increased during treatment. There was a significant relationship between acute renal failure (ARF) and hypokalemia at the time of admission (*P*<0.001), potassium loss during treatment (*P*<0.001), acidosis (0.005), and cholera-related diarrhea (0.05).

**Conclusion:** The high prevalence of hypokalemia in these patients as well as potassium loss during treatment indicates insufficient level of potassium in the therapeutic solutions. Mild hyponatremia in most patients highlights the need for isotonic solutions to treat dehydration.

**Implication for health policy/practice/research/medical education:**

In a historical cohort study on 121 patients who were hospitalized due to acute diarrhea, we found the high prevalence of hypokalemia in these patients as well as potassium loss during treatment which indicates insufficient level of potassium in the therapeutic solutions. Mild hyponatremia in most patients highlights the need for isotonic solutions to treat dehydration.

## Introduction

Acute diarrhea has a high prevalence rate all across the world and about 5%-20% of the world population are affected by diarrhea each year (1). Acute diarrhea causes more than 5-8 million deaths per year (2). Acute diarrhea is the main cause of protein–calorie malnutrition in developing countries (3). In developed countries, like the United States, acute diarrhea leads to very significant economic losses, including 250 000 hospitalizations and almost 8 million visits to physicians each year (4-6). Acute diarrheal diseases are a leading cause of morbidity and mortality in Asia, Africa, and Latin America and are responsible for 4-6 million deaths annually (7,8). The leading causes of mortality from acute diarrhea are dehydration, electrolyte disorders, and their associated complications (9,10). Cholera is an important cause of severe acute diarrhea (11). Untreated severe acute cholera-related diarrhea can lead to up to 50% mortality (12). Approximately 5.5 million cholera-related diarrheas occur annually worldwide and about 100 000 of patients die (12,13). A new subgroup of cholera-related diarrhea epidemic has spread from India to other parts of the Middle East and Asia that can be considered as the main cause of cholera-related diarrhea epidemics in these areas in recent years (14). The leading cause of death in severe acute cholera-related diarrhea, like other types of severe acute diarrhea, is dehydration and water and electrolytes disorders (15).

## Patients and Methods

In this study, 121 patients with severe acute diarrhea who had been hospitalized in eight large hospitals in Tehran including Shahid Labbafinejad, Ayatollah-Taleghani, Hafte-Tir, Firoozgar, Loghman-Hakim, Bu Ali, Imam-Khomeini and Amir-Alam were enrolled in the study. These hospitals are located in different parts of Tehran, including north, east, west, and south of Tehran. The records of these patients were studied via a historical cohort approach. Initially, all patients who were admitted to hospitals with severe acute diarrhea (loss of >10% of body weight due to dehydration or those who were not able to drink water or were affected by vomiting and impaired consciousness) and received a single protocol for the treatment, i.e. the WHO guideline, were enrolled into this study. We recorded all patients’ data including age, sex, cause of diarrhea, residential location, duration of hospitalization, date of hospitalization, and changes in potassium, sodium, calcium, creatinine (Cr), and pH on admission and during treatment. To avoid the effects of confounding factors in the study, patients with underlying diseases such as diabetes, immune suppressive therapy –malignant cancer –, chronic renal failure, and those with heart and brain and liver diseases were excluded from the study. In addition, patients who had been admitted with severe acute diarrhea and received a treatment protocol dissimilar to other patients were excluded from the study at the early stages. In addition, cholera was confirmed by TCBS transport carrier.

### Ethical issues

The research was conducted as retrospective study and followed the tenets of the Declaration of Hel­sinki. All information remains confidential.

### Statistical analysis

After entering data into master sheet, they were entered into an Excel database and were analyzed using SAS 2000 software and *P* value less than 0.05 was considered as statistically significant. The relationships between acute renal failure (ARF) and need for dialysis and the frequency of hypokalemia, hyponatremia, and hypocalcemia acidosis were analyzed through chi-square, Wilcoxon signed ranks, and fisher’s exact tests. Electrolyte disorders and pH were assessed on admission and after treatment.

## Results

Of 121 patients, 47.1% were female. Of all, 98 persons (81%) were living in Tehran and the rest of patients (19%) were residents of other cities of the country. A total of 28 patients (23.3%) were hospitalized for less than three days, 51 patients (42.5%) were hospitalized for three to seven days, and 41 patients (34.2%) were hospitalized for longer than seven days. Concerning the etiology of the disease, 59 cases (48.8%) were due to cholera, 32 cases (26.4%) due to amebiasis, five cases (4.1%) due to shigellosis; the main cause of diarrhea was not found in and the rest of the 25 patients (20.7%).

At the time of admission, 82 patients (67.8%) had hyponatremia (plasma Na < 137 mEq/L) and only 7 patients (5.7%) developed hypernatremia (plasma Na >143 mEq/L). Moreover, 63 patients had mild hyponatremia (120 <plasma Na <137 mEq/L), and there was only one case of severe hyponatremia (plasma Na <120 mEq/L). Of all, 41 patients (33.88%) had hypokalemia on admission. They had plasma potassium less than 3.5 mEq/L, and only three persons (2.4%) were diagnosed with hyperkalemia. Furthermore, 23 (56.1%) of patients with mild hypokalemia were affected by severe form (3 < plasma K <3.5 mEq/L) and 18 persons (43.9%) suffered from its severe form (plasma K <3 mEq/L).The low potassium level was more prevalent than the severe hyponatremia (43.9% vs. 1.4%).

Of all, 23 patients had hypocalcemia (plasma Ca <8.5 mg/dL) and only one person had hypercalcemia (plasma Ca> 10.5 mg/dL). Moreover, in 28 patients (23.9%) serum Cr level was higher than 1.5 mg/dL on admission that indicates that they were affected by ARF. Of these patients, 13 persons (46.4%) were affected by the mild form of the increase in the serum Cr (1.5 <plasma Cr <3 mg/dL) and 15 patients (53.6%) had a plasma Cr level above 3 mg/dL. In addition, 63 patients (56.75%) had acidosis (pH <7.34) and 16 patients (14.41%) had alkalosis (pH > 7.43). Of all patients with acidosis, 23 patients had mild acidosis (7.2 <plasma pH <7.34) and 15 patients (31.24%) had severe acidosis (pH <7.2). Of the 28 patients with ARF 23.8% required dialysis during hospitalization.

Of patients with hypokalemia who were treated according to the WHO’s protocol, the plasma potassium of 32 patients (78%) was not corrected during hospitalization and they even suffered from increased loss of potassium; as a result, they needed additional measures. Of patients with hyponatremia who were treated according to the WHO’s protocol, all cases of disorders were corrected. In 21% of patients with acidosis, the problem was not corrected, worsened, and further measures were needed during the treatment. Of patients with hypocalcemia who were treated according to the WHO’s protocol, all cases of disorders were corrected. Cholera-related diarrhea had a significant relationship with decreased plasma potassium on admission (*P* < 0.001), hyponatremia on admission (*P* < 0.001), no response to hydration according to WHO’s protocol (*P* < 0.001) and the need of patients for hemodialysis. Plasma potassium loss during treatment was significantly associated with Acute kidney injury (AKI) (*P* < 0.001). Cholera-related diarrhea was significantly associated with AKI (*P* < 0.05). However, there was no significant relationship between AKI and duration of hospitalization, acid-base disorders, and hypocalcemia.

## Discussion

The high prevalence of cholera-related diarrhea, which was observed in 121 of the studied patients who suffered from acute diarrhea, indicated the severity of dehydration in this group of patients (16). The high prevalence of hypokalemia was in line with the results of previous studies (17). Half of the patients had severe hypokalemia and as it was associated with the high prevalence of acidosis, which highlights the severity of hypokalemia in these patients (18,19).

Despite treating all patients according to a single WHO’s protocol which includes potassium, 78% of patients were affected by loss of potassium or uncorrected plasma potassium levels and required additional measures. Although the correction of acidosis can cause loss of plasma potassium levels during treatment, the observations were indicative of the inability of these protocols for the correction of plasma potassium levels (20,21).

High prevalence of hyponatremia in these patients and low prevalence of hypernatremia indicates the prevalence of watery diarrhea in most patients and indicates the low alertness of patients to drink water (22,23). Most of the patients had mild hyponatremia, which implies the need for the isotonic oral and injectable fluids (24). The high prevalence of hypocalcemia shows the need for precise measurement of symptoms of hypocalcemia in these patients (15). About one-fourth of patients were affected with AKI which indicated the severity of dehydration (18,24). On the other hand, due to the significance of hypokalemia on admission, potassium loss during treatment, and the association between acidosis and cholera-related diarrhea and AKI and the need for hemodialysis, it is necessary to provide early and appropriate treatment for these patients (25).

## Conclusion

In conclusion, the results of this study shows the need for further and more precise researches on the type and composition of the liquids administered for the treatment of acute diarrhea and it recommends researches on potassium and alkali levels in these solutions.

## Limitations of the study

Low proportion of patients was a limitation of our study.

## Acknowledgments

This study was extracted from thesis of Alireza Soleimani in Shahid-Baheshti University of Medical Sciences.

## Authors’ contribution

All authors participated equally in all stages of the study.

## Conflicts of interest

The authors declare no conflict of interest.

## Ethical considerations

Ethical issues (including plagiarism, data fabrication, double publication) have been completely observed by the authors.

## Funding/Support

None.
